# Mesenchymal Stem Cell Therapy for a Better Prognosis of Heart Failure: A Systematic Review and Meta-Analysis of Randomized Controlled Trials

**DOI:** 10.7759/cureus.43037

**Published:** 2023-08-06

**Authors:** Gautham Varun Krishna Mohan, Gayathri Tirumandyam, Hema Srikanth Vemulapalli, Jaahnavi Vajje, Hamza Asif, Faraz Saleem

**Affiliations:** 1 Internal Medicine, Tirunelveli Medical College, Tirunelveli, IND; 2 Internal Medicine, Siddhartha Medical College, Dr. YSR University of Health Sciences, Vijayawada, IND; 3 Cardiology, Mayo Clinic, Phoenix, USA; 4 Internal Medicine, Dr. Pinnamaneni Siddhartha Institute of Medical Sciences & Research Foundation, Vijayawada, IND; 5 Pulmonology, Khyber Teaching Hospital, Peshawar, PAK; 6 Internal Medicine, California Institute of Behavioral Neurosciences & Psychology, Fairfield, USA; 7 Internal Medicine, Akhtar Saeed Medical and Dental College, Lahore, PAK

**Keywords:** efficacy, regenerative medicine, cardiovascular disease, treatment, randomized controlled trials, meta-analysis, systematic review, prognosis, heart failure, mesenchymal stem cell therapy

## Abstract

Mesenchymal stem cell (MSC) therapy is a frequently used treatment option for achieving a better prognosis in patients with heart failure (HF). However, due to reported adverse effects, patients are often hesitant to consider this treatment. Consequently, the aim of this systemic review and meta-analysis is to further investigate the effects of MSCs on survival outcomes, hospital readmissions, and left ventricular ejection fraction (LVEF) in individuals with pre-existing HF. We systematically searched PubMed, Web of Science, Embase, and Cochrane Library to review studies published up until July 16, 2023. Risk ratios were generated using the extracted data for all the outcomes except LVEF. The mean difference was generated for LVEF. Sensitivity analysis was performed to investigate heterogeneity, and the risk of bias tool was used to assess the quality of the included studies. Fourteen randomized controlled trials were included in the meta-analysis. Pooled results revealed that the MSC therapy group did not significantly affect the outcomes of cardiovascular death, rehospitalization rate, myocardial infarction, recurrence of HF, and total death when compared to a control group. However, MSC therapy was significantly associated with an increased LVEF (RR = 3.35; 95% CI: 0.79-5.72; p = 0.010; I2 = 95%). Upon sensitivity analysis, MSC therapy was significantly associated with a decreased hospitalization rate (RR = 0.46; 95% CI: 0.34-0.64; p < 0.00001; I2 = 0%). MSC transplantation results in a significantly improved LVEF and rehospitalization rate.

## Introduction and background

Heart failure (HF) is a pathological medical condition that occurs due to the heart failing to pump an adequate amount of blood for the body. This results from either decreased ventricular ejection or the inability of the ventricle to accommodate normal venous return [1]. It has been approximated that HF is the cause of 266,400 deaths annually, and the incidence of HF may increase by 46% (from 2012) until 2030 [2,3]. Furthermore, HF can worsen lifestyle by impairing kidney function and liver function and causing pulmonary hypertension, pulmonary edema, or cardiac arrhythmia [4]. It is thus crucial to focus attention on treatment methods for patients with HF to achieve reduced mortality and control worsening organ function in these individuals.

One such treatment option is the use of mesenchymal stem cells (MSCs). MSCs have been used for many years to improve the prognosis in HF patients [5]. MSCs are a type of stromal cells that can undergo mitosis to replace other degenerated MSCs and can differentiate into a wide variety of other cells. They can thus be easily found in abundance in the bone marrow, adipose tissue, lung tissue, synovial membrane, endometrium, and blood [6].

It has been proposed that the therapeutic effect of MSCs in patients with HF and other cardiovascular diseases may be due to their capability to differentiate into cardiovascular cells, their ability to stimulate the immune system, and their antifibrotic and angiogenetic properties [7]. Through these mechanisms, MSCs have been correlated with a significant improvement in left ventricular ejection fraction (LVEF). LVEF is often used to assess the degree and type of HF (systolic or diastolic). An LVEF of less than 45% is an excellent predictor of increased mortality in patients with cardiovascular disease [8]. Thus, an increase in LVEF with MSCs indicates improved heart function and better survival outcomes in HF patients.

However, due to the emergence of adverse effects with MSCs use, some individuals are reluctant to use them, and thus ongoing research is being conducted regarding their administration. Some studies suggest that MSCs administration can lead to fever, fatigue, sleeplessness, diarrhea, dermatitis, or vascular disorders [9]. Moreover, while existing literature attempts to investigate the association between MSCs and survival outcomes in HF patients, the reported findings are inconsistent.

Some studies suggest that the administration of MSCs in HF patients is safe and advisable, yielding a better prognosis [10-17]. However, other studies indicate no significant difference in survival outcomes or LVEF in HF patients undergoing stem cell therapy [18,19]. Consequently, we conducted a systematic review and meta-analysis to assess the effect of MSC therapy on outcomes among HF patients.

## Review

Methods

This systematic review and meta-analysis adhered to the guidelines outlined in the Preferred Reporting Items for Systematic Reviews and Meta-Analyses (PRISMA) [20].

Search Strategy

**Figure 1:**
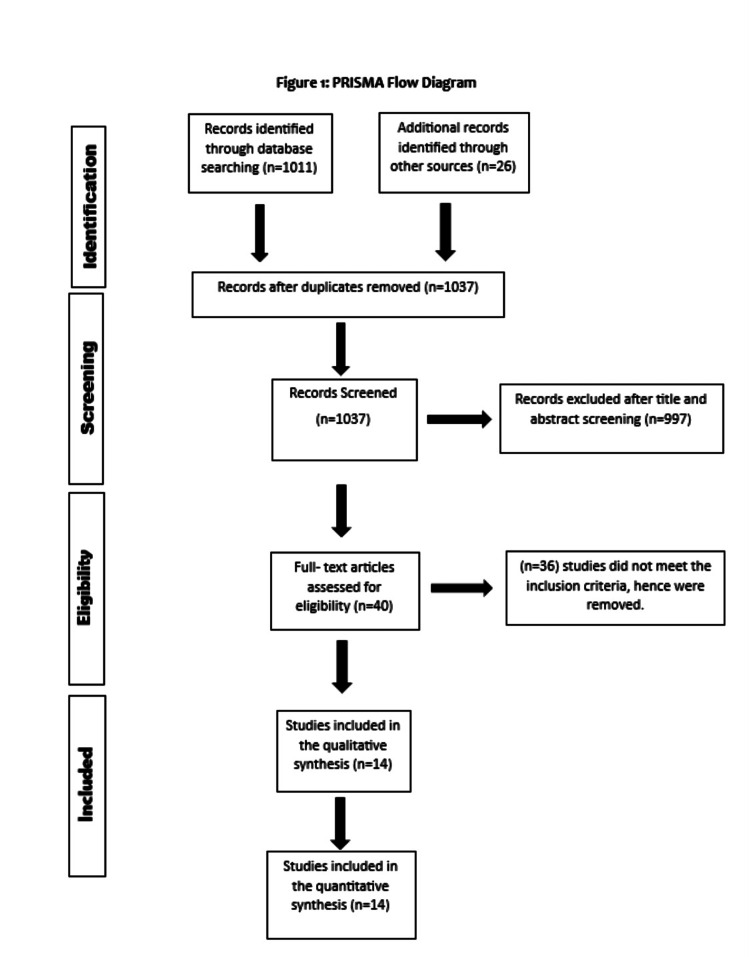
PRISMA flowchart of study selection PRISMA: Preferred Reporting Items for Systematic Reviews and Meta-Analyses.

Two authors conducted independent searches through electronic databases, including PubMed, Web of Science, Embase, and Cochrane Library, to review studies published up until July 16, 2023. Additionally, previous meta-analyses were also reviewed, and relevant studies were extracted. There were no restrictions placed on the geographical area, year of publication, or publication type during the literature review process. The following key terms and words analogous to them were used to search existing literature and identify relevant articles: "mesenchymal stem cell therapy," "mesenchymal stem cells," and "heart failure," along with the Boolean operators "AND" and "OR." Any disagreement regarding the study selection was resolved by consultation with a third author. For further details regarding the search strategy and study selection process, refer to Figure 1.

Study Selection

Studies were included in this meta-analysis based on the following eligibility criteria: (1) studies that were randomized controlled trials (RCTs); (2) studies that examined the effect of MSCs in HF patients; (3) studies that included a control group. All of the included studies were compiled and checked to remove any existing duplicates.

Data Extraction and Quality Assessment

Two reviewers independently extracted relevant data. The following data were extracted: the name of the first author, the year of publishing, publication type, population size, type of MSCs, the method of MSCs administration, mean age of participants, number of males, BMI, New York Heart Association (NYHA) class, and follow-up time. The events/total for all outcomes were also extracted. Our primary outcome was LVEF, while our secondary outcomes were the incidence of cardiovascular death, rehospitalizations, MI, recurrence of HF, and total death. We assessed the quality of all included RCTs using the risk of bias tool [21]. A summary of the results of our quality assessment is available in Appendix A.

Statistical Analysis

Statistical analyses were performed using Review Manager software, version 5.3 (Cochrane Collaboration, Copenhagen, Denmark). The association between MSC therapy and adverse or beneficial outcomes in HF patients was evaluated by collecting relevant data and calculating the corresponding mean difference or risk ratio with a 95% confidence interval (CI) for all outcomes. The results of these analyses were presented in forest plots using a random-effects model. Study heterogeneity was assessed using the I2 statistic. A p-value less than 0.05 was considered statistically significant. Sensitivity analysis was also conducted to address the heterogeneity in the results.

Results

Figure 1 shows the PRISMA flowchart of study selection. Initially, 1,037 possibly pertinent articles in total were found. Duplicates were removed. Finally, the meta-analysis included 14 RCTs that satisfied our inclusion criteria.

The baseline characteristics of the included studies are listed in Table 1. The total number of patients was 1,445, including 83.6% males with a mean age of 41.9 years. The methods for the application of MSC were intracoronary transplantation, intramyocardial injection, and intravenous infusion. The follow-up time for all the included RCTs was more than six months [22-38].

**Table 1:** Baseline characteristics of included studies MSC: mesenchymal stem cell; MPCs: mesenchymal precursor cell; UC-MSCs: umbilical cord-derived mesenchymal stem cells; RCT: randomized controlled trial; HF: heart failure; HFrEF: heart failure with reduced ejection fraction; LV: left ventricle.

Author, year	Study type	Number of participants (MSC group/control group)	Mean age of participants (MSC group/control group)	Number of males (MSC group/control group)	BMI (MSC group/control group)	NYHA class III and IV (MSC group/control group)	Method of stem cell delivery	Type of MSC	Type of HF	Patient population	Control group	Follow-up time
Ascheim et al. (2014) [10]	Multicenter, double-blind, sham-procedure controlled trial	20/10	55.1 ± 15.4/62.2 ± 7.8	17/8	NA	3 and 17/2 and 7	Intramyocardial injection of allogeneic MPCs	Allogeneic MPCs, adult bone marrow-derived mononuclear cells	End-stage heart failure, of either ischemic or nonischemic etiology	Recipients of contemporary left ventricular assist devices (adults with end-stage heart failure)	Cryoprotective medium	Until transplant or 12 months after randomization
Bartolucci et al. (2017) [11]	A phase 1/2 randomized controlled trial	15/15	57.33 ± 10.05/57.20 ± 11.64	12/14	29.12 ± 2.88/29.52 ± 4.00	NA	Intravenous infusion of UC-MSCs	Umbilical cord mesenchymal stem cells	Chronic HFrEF	Patients with stable heart failure and reduced ejection fraction	Placebo	3, 6, and 12 months post-therapy
Bartunek et al. (2013) [12]	Prospective, multicenter, randomized trial	21/24	55.7 ± 10.4/59.5 ± 8.0	20/22	NA	NA	Endomyocardial injection of autologous bone marrow-derived and cardiogenically oriented mesenchymal stem cells	Autologous bone marrow-derived and cardiogenically oriented mesenchymal stem cell	Heart failure of ischemic origin	Heart failure of ischemic origin	Beta-blocker, an angiotensin-converting enzyme inhibitor or angiotensin receptor blocker, and a diuretic	6 months post-therapy, 2 years post-therapy
Bartunek et al. (2017) [18]	Multinational, randomized, double-blind, sham-controlled study	120/151	61.6 ± 8.6/62.1 ± 8.7	107/136	28.2 ± 3.7/28.6 ± 4.4	96 and 1/114 and 1	Cardiopoietic cells delivered endomyocardially with a retention-enhanced catheter	Bone marrow mesenchymal stem cells	Heart failure of ischemic origin	Patients with symptomatic ischemic heart failure	Insertion of an introducer sheath, left ventricular angiography, and pigtail catheter movements	26 and 39 weeks
Bolli et al. (2021) [13]	Double-blind, placebo-controlled, phase II trial	29/32	61.7 ± 6.7/63.1 ± 8.8	27/31	30.4 ± 5.4/30.0 ± 4.4	6/3	Transendocardial injection of MSCs	Autologous bone marrow-derived mesenchymal stromal cells	Heart failure of ischemic origin	Patients with ischemic heart failure	Placebo	12 months
Butler et al. (2017) [36]	Single-blind, placebo-controlled, crossover, randomized phase II-a trial	22 (combined group)	47.3 ± 12.8 (combined group)	13 (combined group)	32.24 ± 7.56 (combined group)	1 (combined group)	Intravenously administered ischemia-tolerant MSCs	Ischemia-tolerant MSCs	Heart failure of non-ischemic origin	Patients with nonischemic cardiomyopathy	Placebo	90 days
Heldman et al. (2014) [14]	Phase 1 and 2 randomized, blinded, placebo-controlled study	19/11	57.1 ± 10.6/60.0 ± 12.0	18/10	NA	2/3	Transendocardial injection of autologous mesenchymal stem cells	Autologous mesenchymal stem cells (MSCs) and bone marrow mononuclear cells	Heart failure of ischemic origin	Patients with ischemic cardiomyopathy and left ventricular (LV) ejection fraction of less than 50%	Placebo	30 days, 1-year post-therapy
Kim et al. (2018) [37]	RCT	14/12	55.3 ± 8.6/57.8 ± 8.9	14/12	NA	NA	Intracoronary delivery of autologous bone marrow mesenchymal stem cells	Autologous bone marrow-derived mesenchymal stromal cells	Congestive HF	Patients with anterior wall ST-segment elevation myocardial infarction	Optimum post-infarction treatment	4 months
Mathiasen et al. (2015) [15]	Randomized, double-blind, placebo-controlled trial	40/20	66.1 ± 7.7/64.2 ± 10.6	36/14	29.8 ± 4.7/28.7 ± 5.3	29/15	Intra-myocardial injections	Autologous bone marrow-derived MSCs	Heart failure of ischemic origin	Patients with severe ischemic heart failure	Placebo	1 month, 3 months, 6 months
Perin et al. (2015) [17]	Phase 2, multicenter, dose-escalation study	45/15	62.2 ± 10.3/62.7 ± 11.2	44/11	29.8 (4.1)/31.3 (9.2)	14 and 0/9 and 0	Transendocardial injection of allogeneic MPCs	Allogeneic MPCs, adult bone marrow-derived mononuclear cells	Heart failure due to left ventricular systolic dysfunction of either ischemic or nonischemic etiology	Patients with chronic heart failure	Mock mapping/injection procedures	13 months post-therapy, 3 years post-therapy
Perin et al. (2023) [16]	Randomized, double-blind, multicenter study	283/282	62.7 ± 10.9/62.6 ± 10.4	222/221	NA	175/178	Transendocardial injection of allogeneic MPCs	Allogeneic MPCs, adult bone marrow-derived mononuclear cells	Heart failure (ischemic or nonischemic)	Heart failure with reduced ejection fraction (HFrEF)	Patients who did not receive stem cell therapy or any placebo transendocardial injections	12 months post-therapy
Xiao et al. (2017) [38]	Randomized comparative study	17/20	51.6 ± 12.2/54.4 ± 11.6	12/14	NA	NA	Intracoronary injection	Bone marrow mesenchymal stem cells	Diastolic HF	Patients with dilated cardiomyopathy	Saline	3 months, 12 months
Yau et al. (2019) [19]	Randomized phase 2 clinical trial	106/53	55.5 ± 12.3/56.9 ± 11.7	94/47	NA	31 and 75/12 and 41	Intramyocardial injection of allogeneic MPCs	Allogeneic MPCs, adult bone marrow-derived mononuclear cells	End-stage heart failure (ischemic or nonischemic)	Recipients of contemporary left ventricular assist devices (adults with end-stage heart failure)	Cryoprotective medium	6 months post-therapy, 1 year post-therapy
Zhao et al. (2015) [35]	RCT	30/29	52.90 ± 16.32/53.2 ± 11.46	24/19	NA	NA	Intracoronary injection of umbilical cord mesenchymal stem cells	Umbilical cord mesenchymal stem cells	Chronic systolic heart failure	Patients with severe systolic HF	Medication	1 and 6 months post-therapy

Primary Outcome: Cardiovascular Death

**Figure 2:**
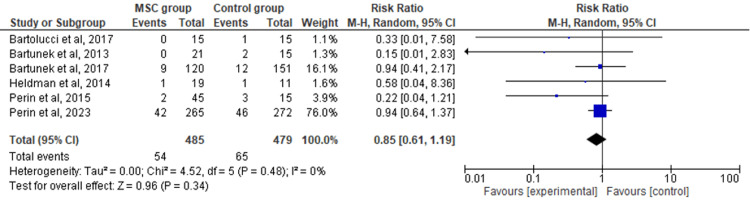
Forest plot for the meta-analysis of cardiovascular death Favors experimental: mesenchymal stem cell (MSC) group.

The random-effects model was used to analyze the primary outcome data. The six included RCTs' pooled estimates indicated that the MSC intervention did not significantly affect cardiovascular death when compared to the control group for HF (RR = 0.85; 95% CI: 0.61-1.19; p = 0.34) (Figure 2). The heterogeneity between the studies was also low (I2 = 0%; heterogeneity p = 0.48).

Secondary Outcomes: LVEF

**Figure 3:**
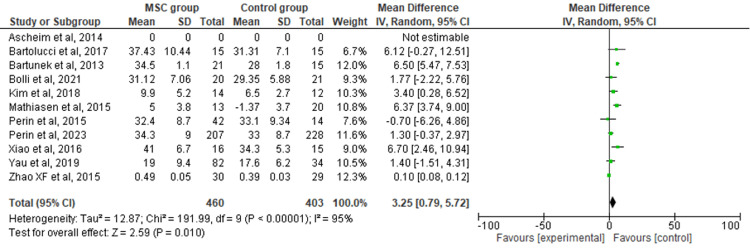
Forest plot for the meta-analysis of left ventricular ejection fraction (%) Favors experimental: mesenchymal stem cell (MSC) group.

The 11 included RCTs' pooled estimates indicated that the MSC intervention was associated with a significantly increased LVEF when compared to the control group (RR = 3.35; 95% CI: 0.79-5.72; p = 0.010; I2 = 95%) (Figure 3). To address the heterogeneity in the results, sensitivity analysis was conducted. The results remained consistent, but the heterogeneity lowered considerably (I2 = 0%; heterogeneity p = 0.48) (Appendix B).

Rehospitalization Rate

**Figure 4:**
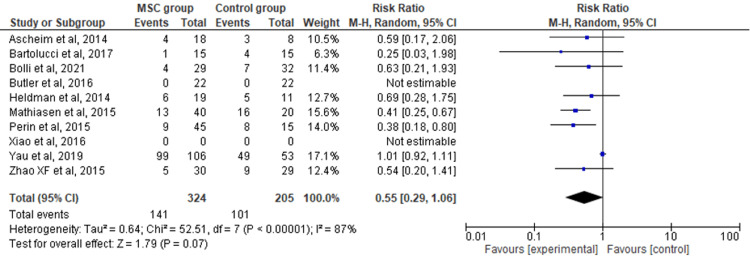
Forest plot for the meta-analysis of the rehospitalization rate Favors experimental: mesenchymal stem cell (MSC) group.

The 10 included RCTs' pooled estimates indicated that there was no significant difference between the MSC intervention group versus the control group for the outcome of rehospitalization rate (RR = 0.55; 95% CI: 0.29-1.06; p = 0.07; I2 = 87%) (Figure 4). Upon conducting sensitivity analysis, the results differed, favoring the MSC therapy group over the control group while the heterogeneity also decreased (RR = 0.46; 95% CI: 0.34-0.64; p < 0.00001; I2 = 0%) (Appendix C).

Myocardial Infarction

**Figure 5:**
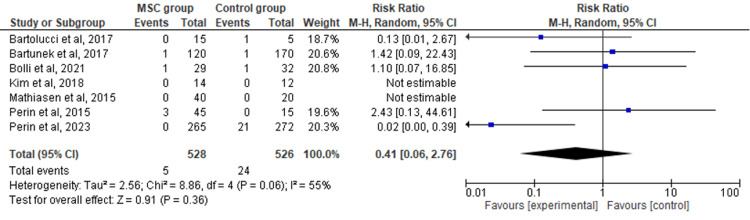
Forest plot for the meta-analysis of myocardial infarction Favors experimental: mesenchymal stem cell (MSC) group.

The seven included RCTs' pooled estimates indicated that the MSC intervention did not significantly affect myocardial infarction when compared to the control group for HF (RR = 0.41; 95% CI: 0.06-2.76; p = 0.36; I2 = 55%) (Figure 5). To address the heterogeneity in the results, sensitivity analysis was conducted. The results remained consistent, but the heterogeneity lowered considerably (I2 = 0%; heterogeneity p = 0.92) (Appendix D).

Recurrence of Heart Failure

**Figure 6:**
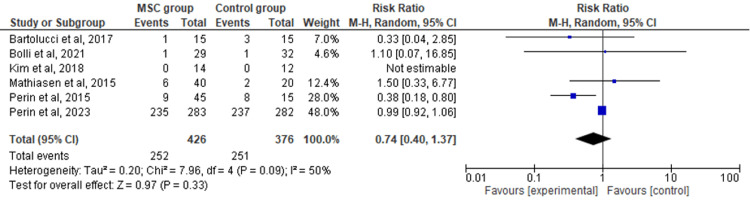
Forest plot for the meta-analysis of the recurrence of heart failure Favors experimental: mesenchymal stem cell (MSC) group.

The six included RCTs' pooled estimates indicated that the MSC intervention did not significantly affect the recurrence of HF when compared to the control group (RR = 0.74; 95% CI: 0.40-1.37; p = 0.33; I2 = 50%) (Figure 6). To address the heterogeneity in the results, sensitivity analysis was conducted. The results remained consistent, but the heterogeneity lowered considerably (I2 = 0%; heterogeneity p = 0.73) (Appendix E).

Total Death

**Figure 7:**
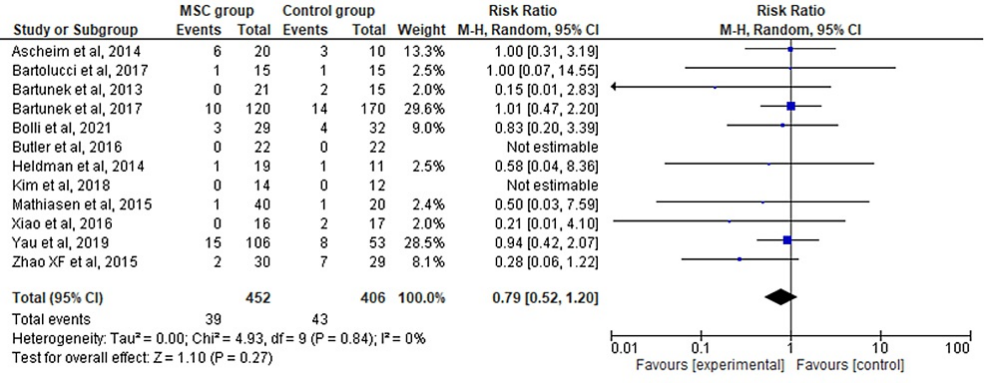
Forest plot for the meta-analysis of total death Favors experimental: mesenchymal stem cell (MSC) group.

The 12 included RCTs' pooled estimates indicated that the MSC intervention did not significantly affect the total death when compared to the control group (RR = 0.79; 95% CI: 0.52-1.20; p = 0.27) (Figure 7). The heterogeneity between the studies was also low (I2 = 0%; heterogeneity p = 0.84).

Discussion

In our meta-analysis to determine the effect of MSC therapy on outcomes among HF patients, MSC therapy did not affect the outcomes of cardiovascular death, rehospitalization rate, myocardial infarction, recurrence of HF, and total death. However, it was observed that MSC therapy was associated with an increased LVEF as compared to the control group. To address the heterogeneity in the results, sensitivity analysis was conducted. The results remained consistent after sensitivity analysis for the outcomes of myocardial infarction, LVEF, and recurrence of HF. Whereas the results differed for the outcome of rehospitalization rate, favoring the MSC therapy group over the control group.

The clinical effect of MSC therapy for HF patients may be attributed to several processes, including regulation of inflammation, decreased myocardial cell death, myocardial fibrosis, enhanced cell differentiation, and neovascularization. Cell recruitment, migration, and adhesion are only a few of the mechanisms that go into integrating MSCs into tissues. Due to their strong potential for migration and positive reaction to serum in HF patients, umbilical cord MSCs may be able to detect biological cues that are responsible for the therapeutic impact of systemic administration. Our meta-analysis indicates that MSC treatment is linked with considerably improved LVEF and decreased rehospitalization rates when compared to control therapies for HF, but with no significant influence on cardiovascular death [11,22,23].

Previous meta-analyses [24-28] have also been conducted to investigate the association between MSC therapy and adverse or beneficial outcomes in HF patients. Similar to our study, Fan et al. [24] (weighted mean difference (WMD) = 5.25), Fu et al. [25] (mean difference (MD) = 9.64), Jayaraj et al. [26] (MD = 4.58), and Shen et al. [28] (MD = 5.66) also found a significantly improved LVEF on the infusion of MSCs. Moreover, parallel to our findings, Fu et al. [25] found no significant effect of MSCs on cardiovascular death, the occurrence of MI, the recurrence of HF, and total death. However, Lalu et al. [27] found no significant correlation between MSC therapy and LVEF in ischemic HF patients. Furthermore, contrary to our results, Fan et al. [24], Fu et al. [25], and Shen et al. [28] found a significant reduction in rehospitalization rates.

A few existing meta-analyses have also investigated the association between MSCs and manifestations of ischemic heart disease, such as acute myocardial infarction [27,29-31]. It is important to note that ischemic heart disease is a prominent causative agent of HF, and thus it is crucial to review the results of these analyses [32]. While all the aforementioned studies showed improved LVEF in patients suffering from ischemic heart disease, no effect on the risk of readmission and mortality was observed. It can thus be concluded that while MSC therapy significantly improves LVEF and heart function in patients with cardiovascular disease, the overall effect on survival outcomes is insignificant.

Strengths and limitations

Although some previous studies [33,34] have solely evaluated the use of a specific subclass of MSCs, our meta-analysis included studies with all types of MSC therapy, whether it was bone marrow or umbilical cord-derived [11,35]. We also included both types of bone marrow-derived stem cells, autologous and allogeneic. Furthermore, while almost all the existing reviews [24-28] have evaluated LVEF and all-cause mortality, only three [24,25,28] of them have reported data on hospital readmission and one [25] of them has reported data on cardiovascular-specific death. Additionally, we included all studies regardless of the method of delivery of MSCs or type of HF. Whereas Fan et al. [24] included only patients with systolic HF, Lalu et al. [27] included patients with ischemic HF. The presence of only RCTs in our analysis ensures that the risk of bias is minimal [10-19,35-38].

However, due to insufficient data available, we have not evaluated the difference in six-minute walking distance (6MWD) and NYHA class post-therapy, which presents an inevitable limitation of our study. Moreover, no subgroup analysis was done to evaluate the effect of the method of introduction of MSCs in patients or the type of MSC administered. Further research is needed to investigate the effect of specific types of MSC therapy in HF patients.

## Conclusions

MSC transplantation results in a significantly improved LVEF. However, due to limited evidence of its effect on survival outcomes and recurrence of HF, more trials should be conducted to investigate the association between this method of treatment and outcomes in HF patients.

## Figures and Tables

**Figure 8:**
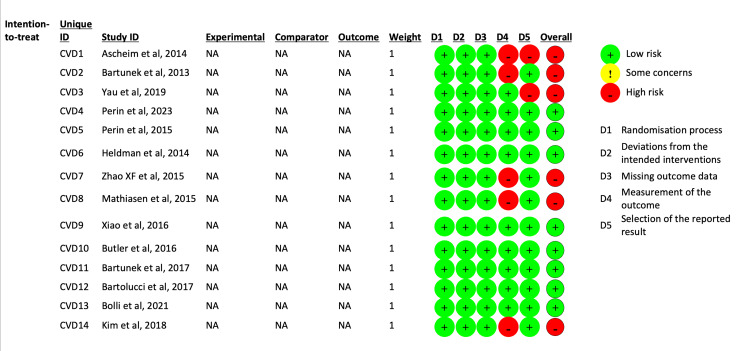
Quality assessment of all the included studies using the risk of bias tool 2.0

**Figure 9:**
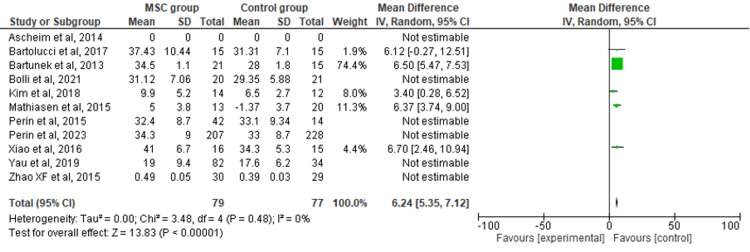
Sensitivity analysis on the outcome of left ventricular ejection fraction MSC: mesenchymal stem cell.

**Figure 10:**
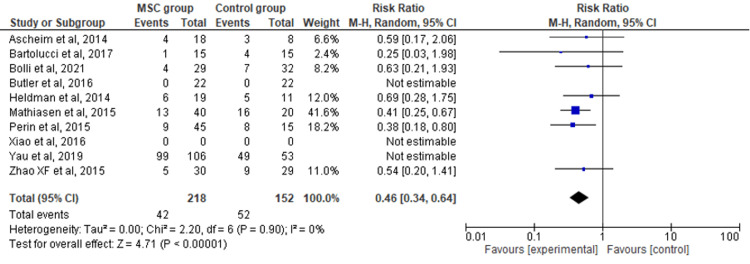
Sensitivity analysis on the outcome of left ventricular ejection fraction MSC: mesenchymal stem cell.

**Figure 11:**
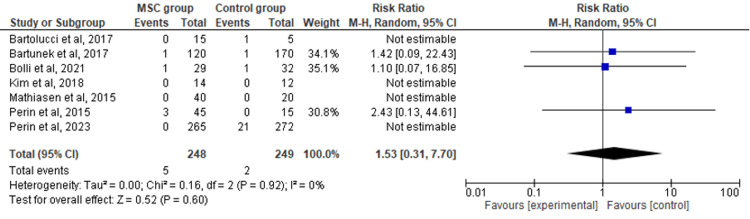
Sensitivity analysis on the outcome of myocardial infarction MSC: mesenchymal stem cell.

**Figure 12:**
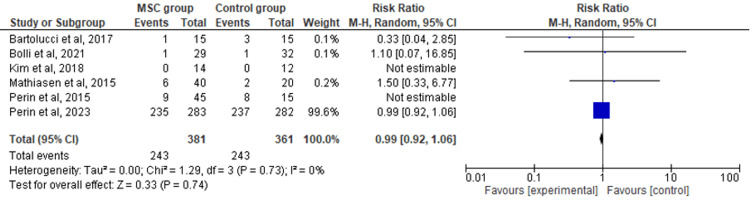
Sensitivity analysis on the outcome of recurrence of heart failure MSC: mesenchymal stem cell.

## Appendices

Appendix A

Appendix B

Appendix C

Appendix D

Appendix E
